# Depression and anxiety among pregnant women during COVID 19 pandemic in Ethiopia: a systematic review and meta-analysis

**DOI:** 10.3389/fgwh.2024.1453157

**Published:** 2024-12-03

**Authors:** Temesgen Gebeyehu Wondmeneh, Mohhamed Wogris

**Affiliations:** Department of Public Health, College of Medical and Health Science, Samara University, Semera, Ethiopia

**Keywords:** depression, anxiety, pregnant women, COVID 19 pandemic, Ethiopia

## Abstract

**Background:**

Coronavirus Disease-19 pandemic had an adverse impact on the mental health of the public worldwide, but the problem is worst among pregnant women due to social distancing policies and mandatory lockdown, including prenatal care services. As a result, the prevalence of depression and anxiety could increase during the pandemic, particularly among pregnant women. Thus, the purpose of this review is to determine the magnitude of depression and anxiety and contributing factors among pregnant women during the pandemic in Ethiopia.

**Methods:**

Web of Science, Since Direct, PubMed, Google Scholar, and African Journals Online were the electronic databases searched, the Preferred Reporting Items for Systematic Reviews and Meta-Analyses (PRISMA) reporting guidelines were followed in this review. The Newcastle-Ottawa Critical Appraisal Checklist was used to assess the quality of the included studies. A predefined data extraction sheet developed in Excel was used to extract the data. The pooled prevalence of anxiety and depression was determined by a random effect model meta-analysis.

**Results:**

4,269 and 1,672 pregnant women were involved in depression and anxiety studies, respectively. The pooled prevalence of depression and anxiety among pregnant women during the COVID-19 pandemic in Ethiopia was 24.7% (95% CI: 18.52–30.87) and 35.19% (95% CI: 26.83–43.55), respectively. Single marital status (AOR = 2.22, 95% CI: 1.07–3.37), poor social support (AOR = 2.7, 95% CI: 1.06–4.35), unplanned pregnancies (AOR = 2.17, 95% CI: 1.34–3.0), and unsatisfied marital status (AOR = 2.16, 95% CI: 1.17–3.14) were risk factors for depression. Violence against intimate partners (AOR = 2.87, 95% CI: 1.97–3.77) and poor social support (AOR = 1.98, 95% CI: 1.24–2.71) were risk factors for anxiety.

**Conclusion:**

One-fourth and nearly one-third of pregnant women had depression and anxiety, respectively, during COVID-19 pandemic in Ethiopia. Single or unsatisfied marital status and unplanned pregnancies were risk factors for depression. Poor social support was significantly associated with depression and anxiety. Pregnant women who experienced violence against intimate partners had higher anxiety. After COVID-19 pandemic, mental health interventions are essential for reducing depression and anxiety.

**Systematic Review Registration:**

https://www.crd.york.ac.uk/prospero/display_record.php?RecordID=527148, PROSPERO (CRD42024527148).

## Background

Mental health is one of the major health indicators that contribute to significant morbidity (1). The most common forms of mental illness are anxiety and depression disorders, which are highly comorbid and together include the more general heading of internalizing disorders (2). Since the onset of Coronavirus Disease-19 (COVID-19) pandemic, the mental health of people has been negatively affected, especially those from vulnerable groups (3). Globally, the prevalence of depression was 32.60% for pregnant and postpartum women during COVID-19 pandemic. The rate was 31.5% for pregnant women and 27.64% for postpartum women (4). The pooled prevalence of depression and anxiety in pregnancy during COVID-19 pandemic was 25.6% and 30.5%, respectively (5). The prevalence of antenatal depression in Africa was 26.3% (6). In Kenya, depression and anxiety were 16.2% and 6.6% among pregnant women, respectively (7). In Ethiopia, the prevalence of depression ranged from 21.3% to 27.9% (8–10). Among pregnant and lactating mothers in Ethiopia, the pooled prevalence of anxiety and depression during COVID-19 pandemic were 33% and 27%, respectively (11).

Financial problem was associated with depressive and anxiety disorder (12). In low-income countries, anxiety disorder was high (13). Lifting people out of poverty improved mental health problems (14). Increased social distance was associated with increased symptoms of anxiety (15). Cultural differences exist in anxiety disorders (16). A person's social concerns need to be considered in light of their cultural, racial, and ethnic background in order to adequately assess the degree and expression of social anxiety as well as its impact on anxiety (17). Low social support was a risk factor for depression, anxiety, and self-harm during pregnancy (18). Genetics, social networks, societal levels, and adverse childhood experiences were risk factors for depression (19).

Understanding the effect of COVID-19 pandemic on psychological changes in pregnant women is essential to preventing its negative impacts and unexpected consequences (4). It is evident that depression, anxiety, and associated risk factors among pregnant women were not examined at the national level during COVID-19 pandemic in Ethiopia. The aim of the current study was therefore to determine the pooled prevalence of depression and anxiety among pregnant women during COVID-19 pandemic in Ethiopia. The present study was the first to examine the depression and anxiety experienced by pregnant women during COVID-19 pandemic at the national level in Ethiopia.

## Methods

### Reporting and registration

This study was registered in PROSPERO with an ID of CRD42024527148. The Preferred Reporting Items for Systematic Reviews and Meta-Analyses (PRISMA) reporting guidelines were followed to report this review (20) (Supplementary File S1).

### Searching strategies

#### Search strategy

A comprehensive electronic search of studies was carried out on the databases of Web of Science, Science Direct, PubMed, Google Scholar, and African journals online from June 4 to 17, 2024. The search strategies were then updated from November 5 to 10, 2024. For PubMed, the MeSH terms used were depression, anxiety, “pregnant women,” and Ethiopia. Database-specific subject headings were used for the other electronic databases that were linked to the terms and keywords listed in PubMed. For additional studies, references to relevant studies were searched. The search was restricted from the publication years of January 1, 2020, until June 17, 2024, with consideration of the onset of the COVID-19 pandemic. The details of the search strategies were provided in the Supplementary File (Supplementary File S2).

#### Eligible criteria

The inclusion criteria were as follows:

• Study design: observational studies
• Study type: both published and unpublished
• Study population: pregnant women
• Language: articles published in English
• Outcome: studies that reported either the prevalence of depression or anxiety or studies that have enough data to calculate the percentage of depression and anxiety.
• Study site and period: studies conducted in Ethiopia from January 1, 2020, to June 18, 2024.

The exclusion criteria were as follows:

• Studies dealing with depressive or anxiety disorders in nonpregnant women, such as postpartum periods.
• Studies conducted on HIV-positive pregnant women were excluded because HIV-positive women are a psychologically different target population from pregnant women without HIV-positive.
• Studies deal with generalized anxiety among pregnant women because generalized anxiety is characterized by chronic and persistent worry (21), which is different from normal anxiety that is usually short-term and related to a stressor response to some event.
• Studies without a clear outcome
• Qualitative studies, editorials, letters, reviews, commentaries, and interventional studies were excluded.

### Outcome measurement

The primary outcome of this review was to determine the pooled prevalence of depression and anxiety during the COVID-19 pandemic in pregnant women using primary study data. Two parameters were required from primary studies to calculate the prevalence of depression and anxiety: the number of pregnant women with depression and anxiety as well as the total sample sizes. The prevalence was calculated by dividing the total number of pregnant women with depression by the total sample size, and the prevalence for anxiety was calculated by dividing the total number of pregnant women with anxiety by the total sample size and multiplying them by 100.

### Study selection

Endnote X8.1 software was used to remove duplicate studies. Then two authors (TGW and MW) independently screened the article title and abstract. The full-text articles were retrieved after the screening of titles and abstracts. Two authors (TGW and MW) further evaluated the full-text article's eligibility for final inclusion. Disagreements were settled through dialogue and scientific consensus between the two authors (TGW and MW).

### Data extraction process

A standardized data extraction checklist was developed using Microsoft Excel. The data were extracted independently by two authors (TGW and MW). Name of the first author, study year, study region, study subjects, sample size, diagnostic tools, cases with anxiety and depression, and prevalence of anxiety and depression were the extracted data. There were no discrepancies between the two authors during data extraction. The extracted data were cross-checked interchangeably by the two authors (TGW and MW). The details of data extraction were provided in the Supplementary File (Supplementary File S3).

### Quality assessment of studies

Critical appraisal was carried out in the included studies by two authors (TGW and MW) independently using the Newcastle Ottawa Critical Appraisal Checklist for adapted cross sectional studies (22). Discussion and scientific consensus were used to settle disagreements between the two authors. The tool consists of three domains: selection with a maximum of 5 points (representativeness of sample*, sample size justification*, non-respondents*, ascertainment of exposure**), comparability with a maximum of 2 points (confounding control**), and outcome assessment with a maximum of 3 points (independent outcome assessment** and statistical test*). The scores were summed up and changed to percentages. When the scores were added up, the minimum score was zero and the maximum score was 10. In this systematic review, studies with quality scores higher than 50% were included.

### Data synthesis and statistical analysis

The relevant information from each original study was extracted using a predefined spreadsheet format developed from Microsoft Excel. Then the data were imported into STATA 15 statistical software for analysis. The random effect model meta-analysis was used to measure the pooled estimates due to the existence of heterogeneity (23). The pooled prevalence of depression and anxiety was computed. The heterogeneity of effect size was examined using the Q statistic and the I^2^ statistic (24). The Q-test determines whether the observed effect size is significantly different from one another than expected by chance. The I^2^ values of 0, 25%, 50%, and 75% were interpreted as no, low, medium, and high heterogeneity, respectively. Significant heterogeneity was taken into account in the current meta-analysis when the I^2^ value was greater or equal to 50%, with *p*-value <0.05. Sensitivity and sub-group analyses were used to investigate potential sources of heterogeneity (25). To identify associated factors, the pooled effect was computed from odds ratios. The results of the meta-analysis were presented in forest plots and tables with the support of a written explanation.

### Ethics statement

Ethical approval was not required for this study since it only uses data from published studies.

## Results

### Search results

**Figure 1:**
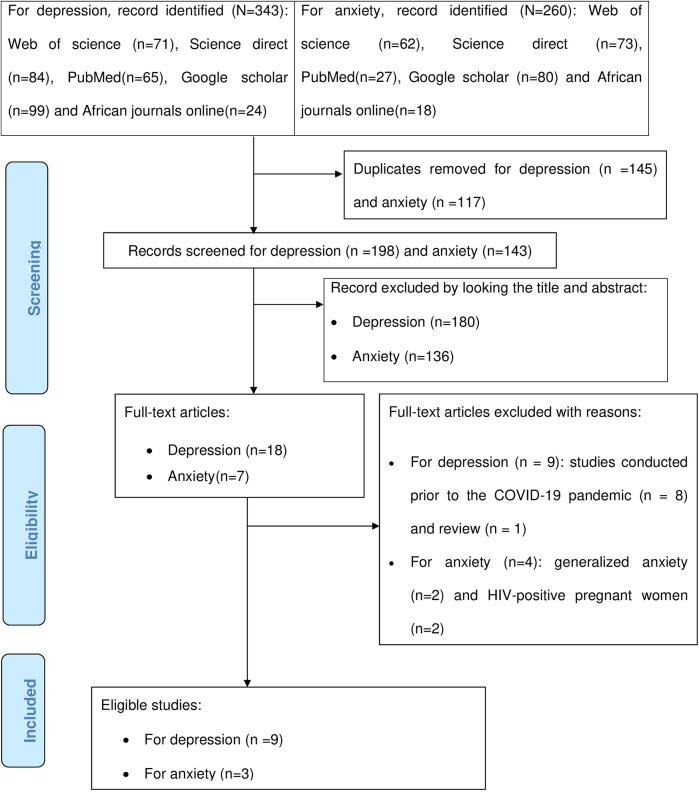
Shows the PRISMA flow chart for the selection of studies for systematic review.

For the initial search, a total of 343 and 260 articles were retrieved for depression and anxiety, respectively. About 145 and 117 duplicate articles were removed for depression and anxiety, respectively. Irrelevant articles excluded based on abstracts and titles for depression and anxiety were 180 and 136, respectively. We excluded nine full-text articles on depression based on the following criteria: Eight studies were done before the COVID-19 pandemic, and one study was a review. Four full-text articles on anxiety were excluded for the following reasons: Two were generalized anxiety and two on HIV-positive pregnant women. Finally, nine eligible studies were found for depression, and three eligible studies were found for anxiety (Figure 1).

### Study characteristics

Nine studies were found, with all of them providing data on depression. Three studies simultaneously reported both depression and anxiety (26–29), and the remaining six studies recorded only depression (30–34). In the Amhara and Oromia regions, six and three studies were conducted, respectively. All the included studies were cross-sectional studies. Six out of eight studies were carried out in 2021. The largest sample size was 858 in a study reporting data on both depression and anxiety (29). For depression, the smallest sample sizes were 329 (30). The minimum prevalence of depression and anxiety were 11.2% and 29.4% (29), respectively. The maximum prevalence of depression and anxiety was 34.1% (32) and 50.3% (27) respectively. Five studies measured depression using EPDS. Three studies used PRAQR to measure anxiety (Table 1).

**Table 1:** Study characteristics.

Author	Study year	Region	Study subjects	Sample size	Outcome	Cases (%)	Tools
Takelle GM, et al. (31)	2022	Amhara	Pregnant women	473	Depression	91 (19.2)	EPDS
Sewnet AN, et al. (32)	2021	Amhara	Pregnant women	422	Depression	144 (34.1)	DASS
Ahmed SJ, et al. (30)	2021	Oromia	Pregnant women	329	Depression	109 (33.1)	EPDS
Anbesaw T, et al. (33)	2021	Amhara	Pregnant women	451	Depression	84 (18.6)	EPDS
Seid J, et al. (34)	2022	Amhara	Pregnant women	552	Depression	178 (32.2)	DASS
Oljira L, et al. (26)	2022	Oromia	Pregnant women	370	Depression	62 (16.8)	EPDS
Abegaz MY, et al. (27)	2021	Amhara	Pregnant women	408	Depression	115 (28.2)	PHQ-9
					Anxiety	179 (43.9)	PRAQR
Tarafa H, et al. (28)	2021	Oromia	Pregnant women	406	Depression	121 (29.8)	EPDS
					Anxiety	133 (32.7)	PRAQR
Haile TT, et al. (29)	2021	Amhara	Pregnant women	858	Depression	96 (11.2)	PHQ-9
					Anxiety	252 (29.4)	PRAQR

### Quality of included studies

Two reviewers (TGW and MW) assessed the quality of the included studies using the Newcastle Ottawa Critical Appraisal Checklist for adapted cross-sectional studies. Any disagreements between the two authors were resolved through dialog and scientific consensus. For depression, three studies were scored 7 out of 10 (70%), while the remaining six studies scored 10 out of 10 (100%). For anxiety, all of the included studies scored 10 out of 10 (100%). Thus, in both outcomes, all of the included studies score above 50% and were included in the final meta-analysis (Supplementary File S4).

### Pooled prevalence of depression among pregnant women after the onset of COVID-19 pandemic

**Figure 2:**
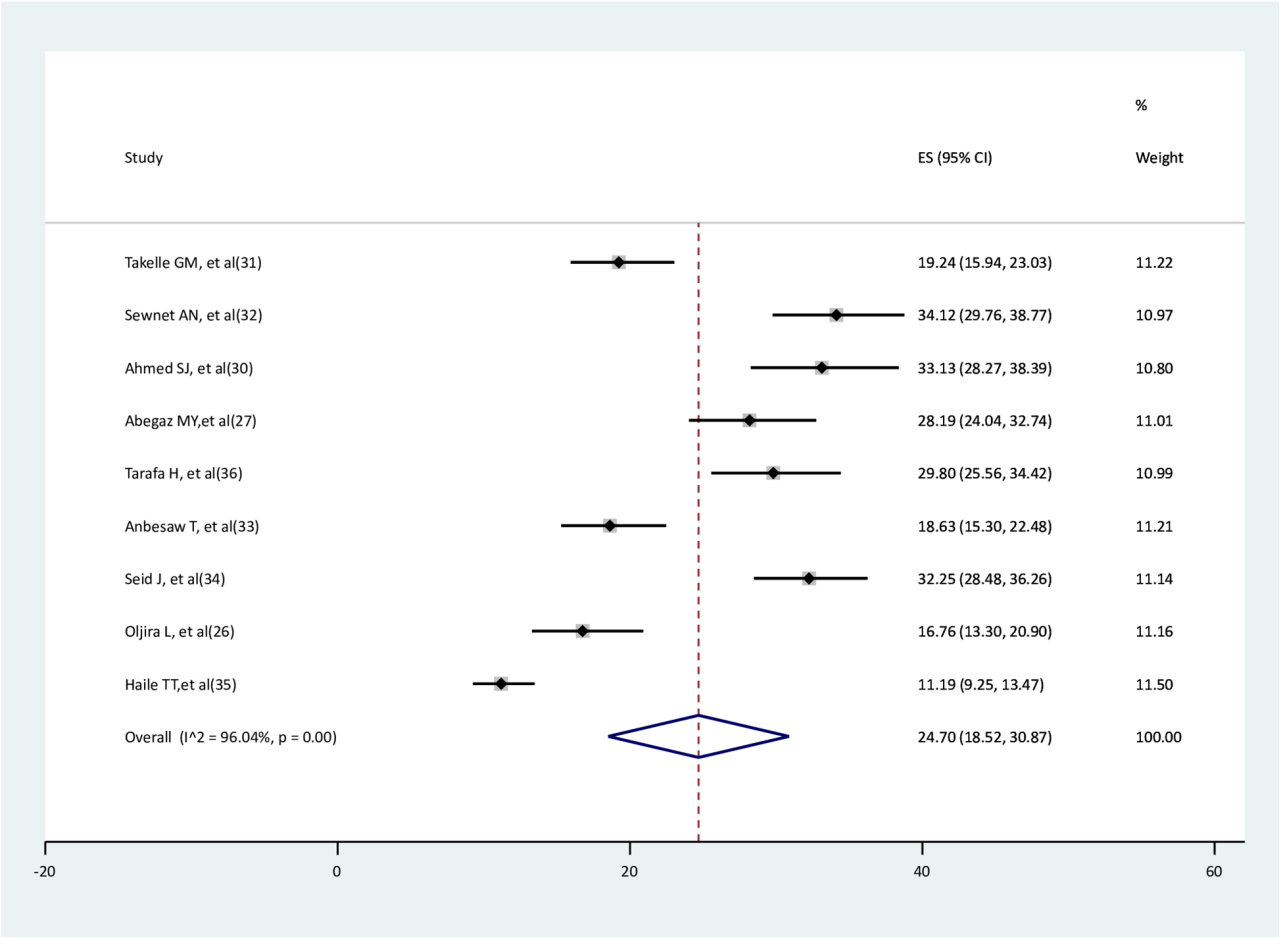
Pooled prevalence of depression among pregnant women.

In this systematic review, 4,269 pregnant women were involved; of them, 1,000 had depression. According to the random effect model meta-analysis, the pooled prevalence of depression among pregnant women was 24.7% (95%CI: 18.52–30.87), with the existence of high heterogeneity (I^2^ = 96.04%, *p* < 0.001) (Figure 2).

### Pooled prevalence of anxiety among pregnant women after the onset of COVID-19 pandemic

**Figure 3:**
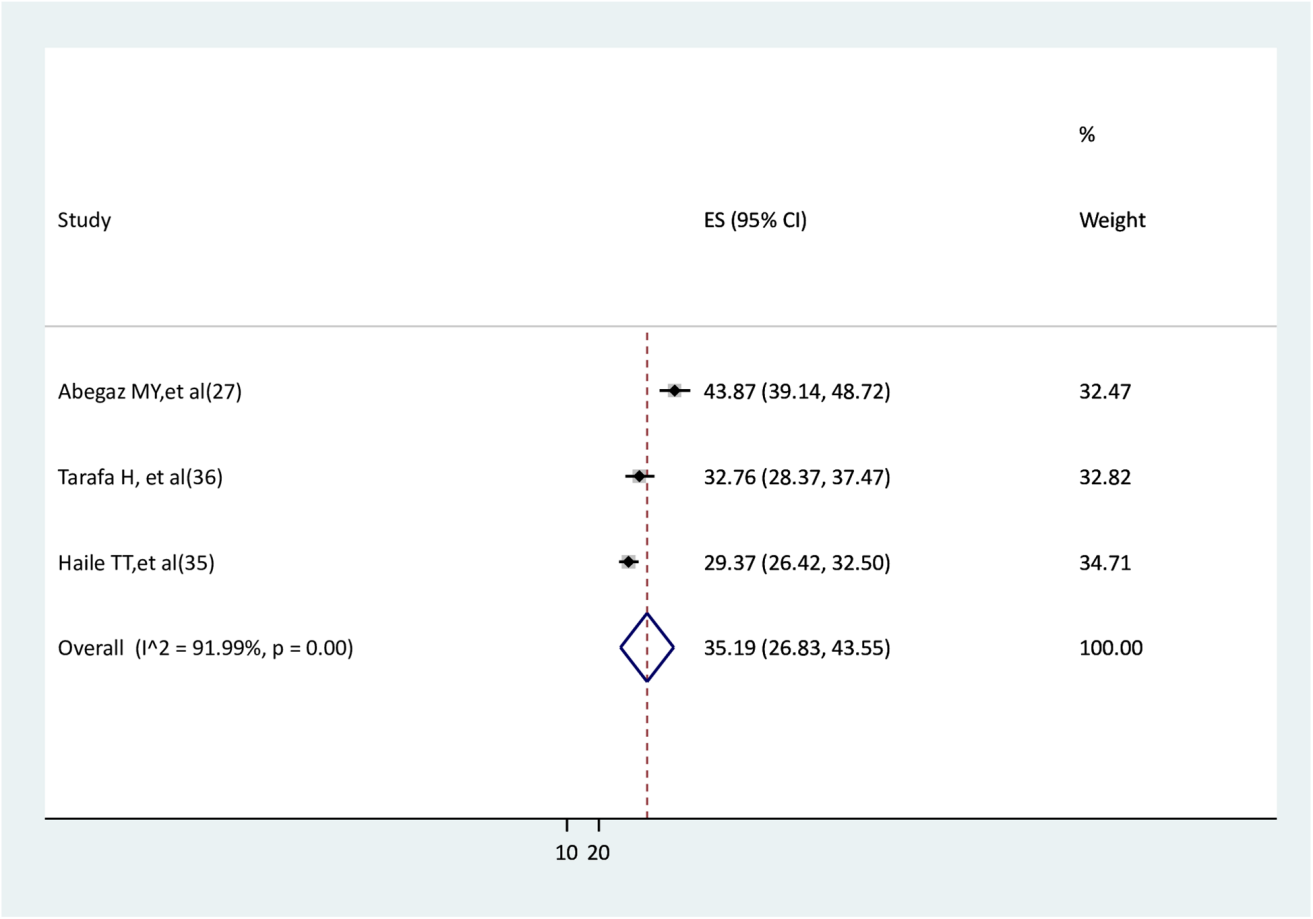
Pooled prevalence of anxiety among pregnant women.

In this review, 1,672 pregnant women were involved; of them, 564 had anxiety. The random effect model meta-analysis revealed that the pooled prevalence of anxiety among pregnant women was 35.19% (95%CI: 26.83–43.55), and high heterogeneity was observed between studies (I^2^ = 91.99%, *p* < 0.001) (Figure 3).

### Subgroup analysis for pooled prevalence of depression

Subgroup analysis based on regions, study year, and measurement tools was conducted for the pooled prevalence of depression. The prevalence of depression in the Oromia and Amhara regions was 26% (95%CI: 16–37) and 24% (95%CI: 16–32), respectively. The pooled prevalence of depression in 2021 and 2022 was 26% (95% CI: 17–34) and 23% (95% CI: 14–32), respectively. The prevalence of depression was 14% (95% CI: 13–16), 23% (95% CI: 17–29), and 33% (95% CI: 30–36) using measurement tools of PHQ-9, EPDS, and DASS, respectively (Table 2).

**Table 2:** Subgroup analysis for pooled prevalence of depression .

Variables	Categories	Prevalence (95%CI)	I^2^, *p*-value	df
Region	Amhara	24% (95%CI:16–32)	96.8%, <0.001	5
	Oromia	26% (95%CI: 16–37)	-	2
Study year	2021	26%(95%CI:17–34)	96.9%, *p* < 0.001	5
	2022	23% (95%CI:14–32)	-	2
Measurement tool	EPDS	23% (95%CI:17–29)	90.7%, *p* < 0.001	4
	PHQ-9	14% (95%CI:13–16)	-	1
	DASS	33% (95%CI:30–36)	-	1

EPDS, Edinburgh postnatal depression scale; PHQ-9, patient health question; DASS, depression anxiety stress scale.

### Sensitivity analysis

**Figure 4:**
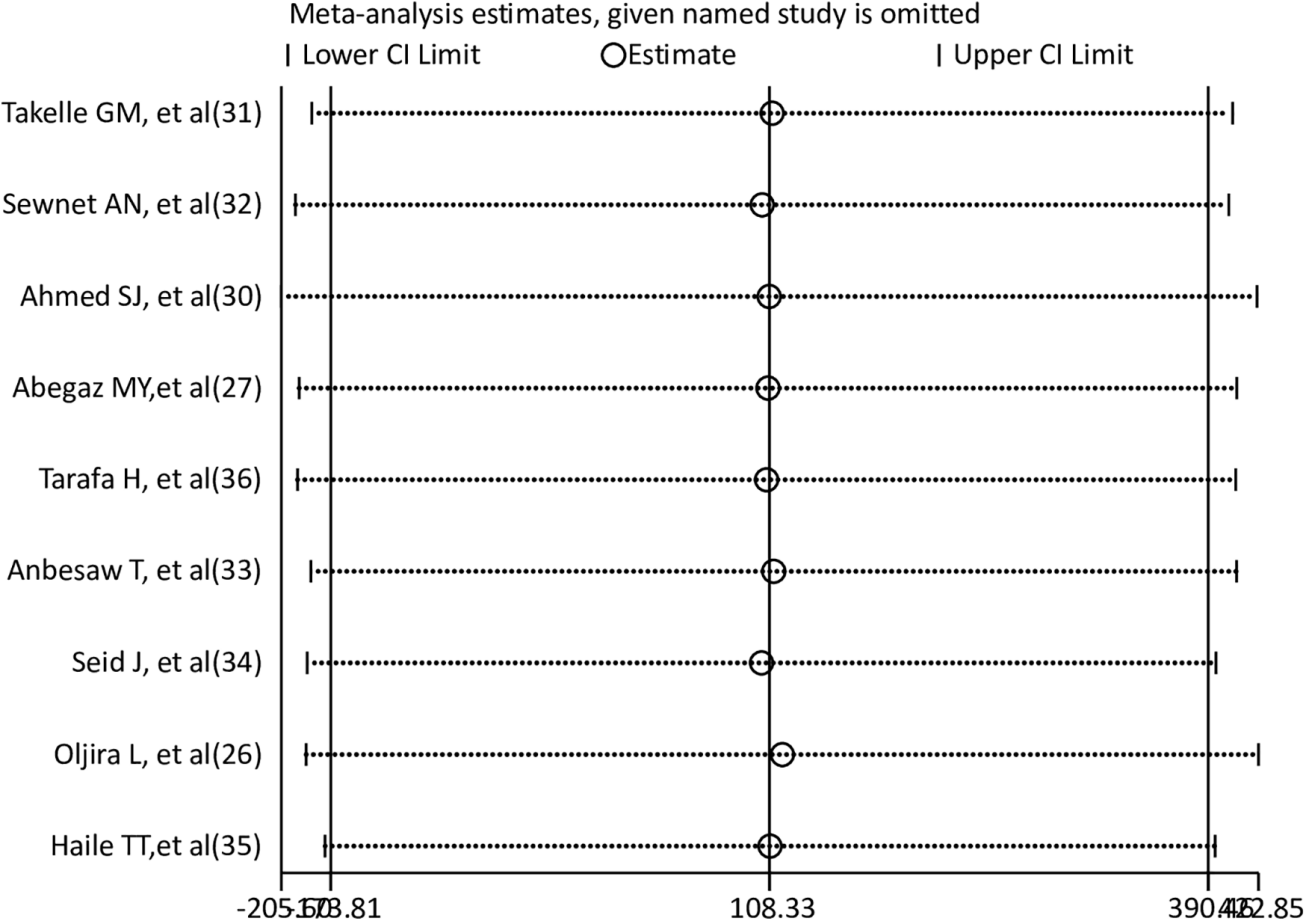
Sensitivity analysis for pooled prevalence of depression among pregnant women.

**Figure 5:**
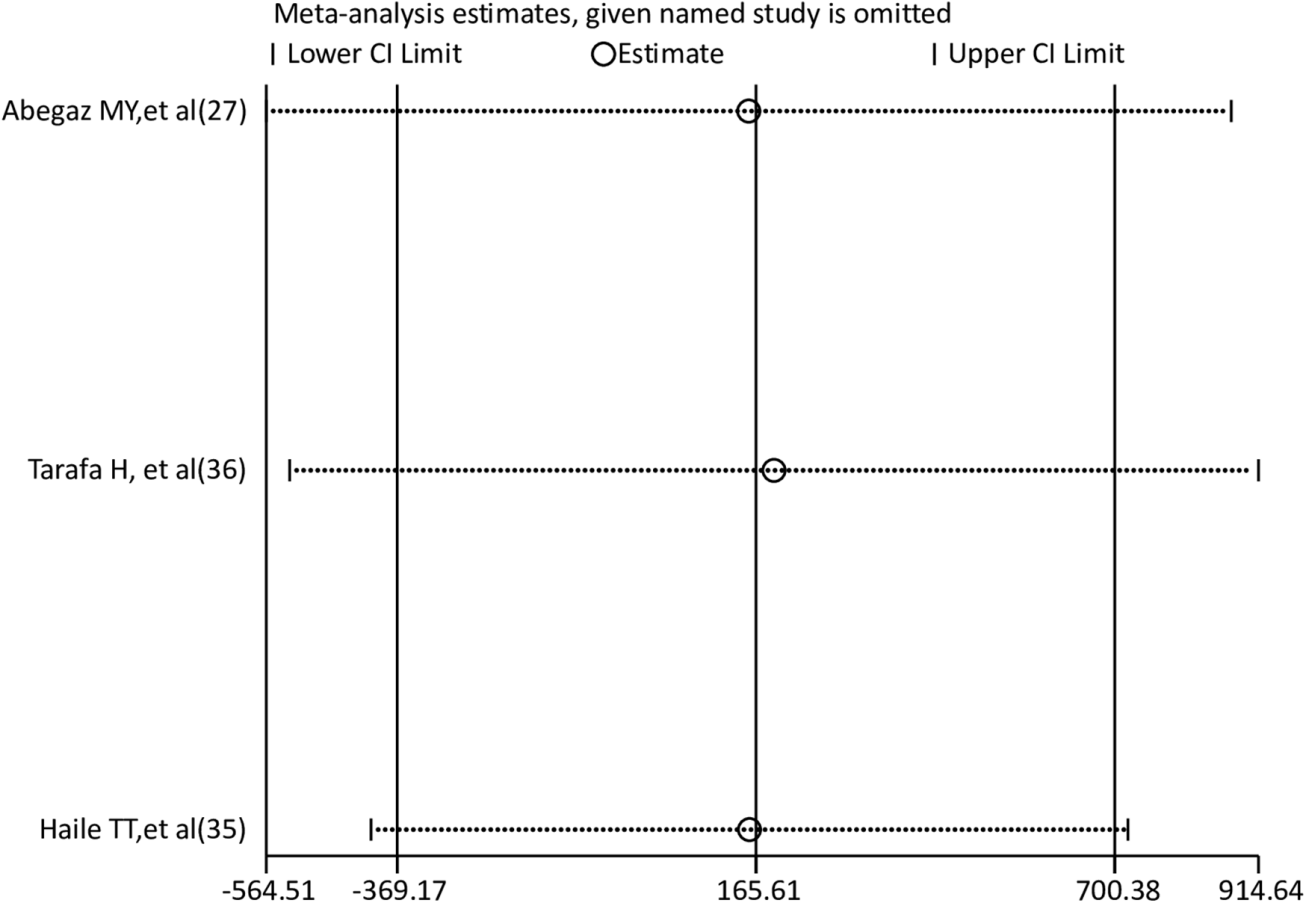
Sensitivity analysis for pooled prevalence of anxiety among pregnant women.

To find the effect of a single study on the pooled prevalence of anxiety and depression among pregnant women, a leave-one-out sensitivity analysis was carried out. The results of the sensitivity analysis showed that the findings for both depression and anxiety were robust and independent of a single study. The sensitivity analysis results were fairly stable or consistent for depression (Figure 4) and anxiety (Figure 5).

## Risk factors for depression

### The association between residence and depression

**Figure 6:**
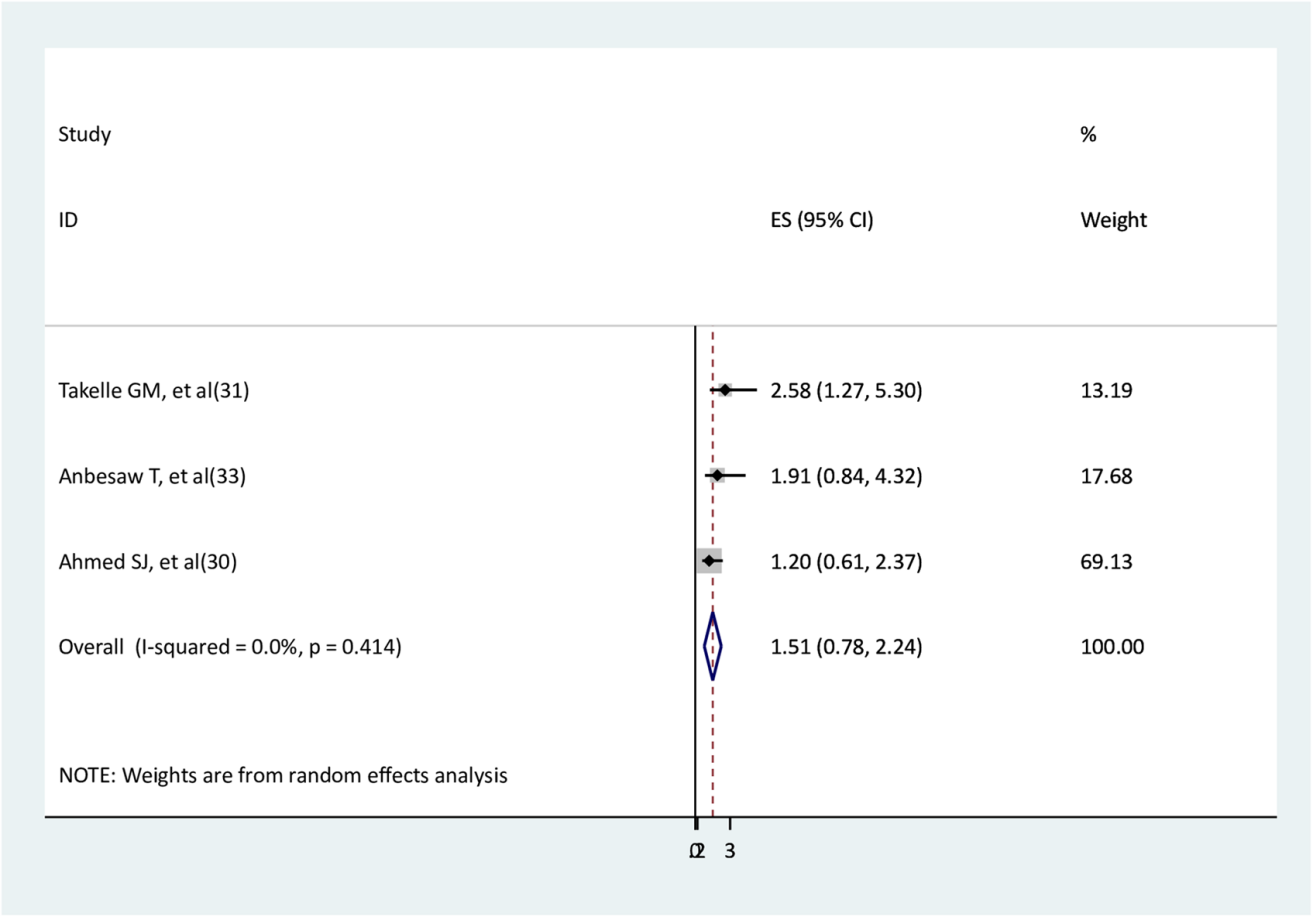
The association between social support and depression.

Three studies (30, 31, 33) were used to assess the association between residence and depression. The findings of one study (31) showed a statistically significant association, whereas the findings of the other two studies (30, 33) revealed no significant association. The results of the meta-analysis indicated that no significant association was observed between being a rural resident and depression (AOR = 1.51, 95% CI: 0.78–2.24) with no heterogeneity (Figure 6).

### The association between marital status and depression

**Figure 7:**
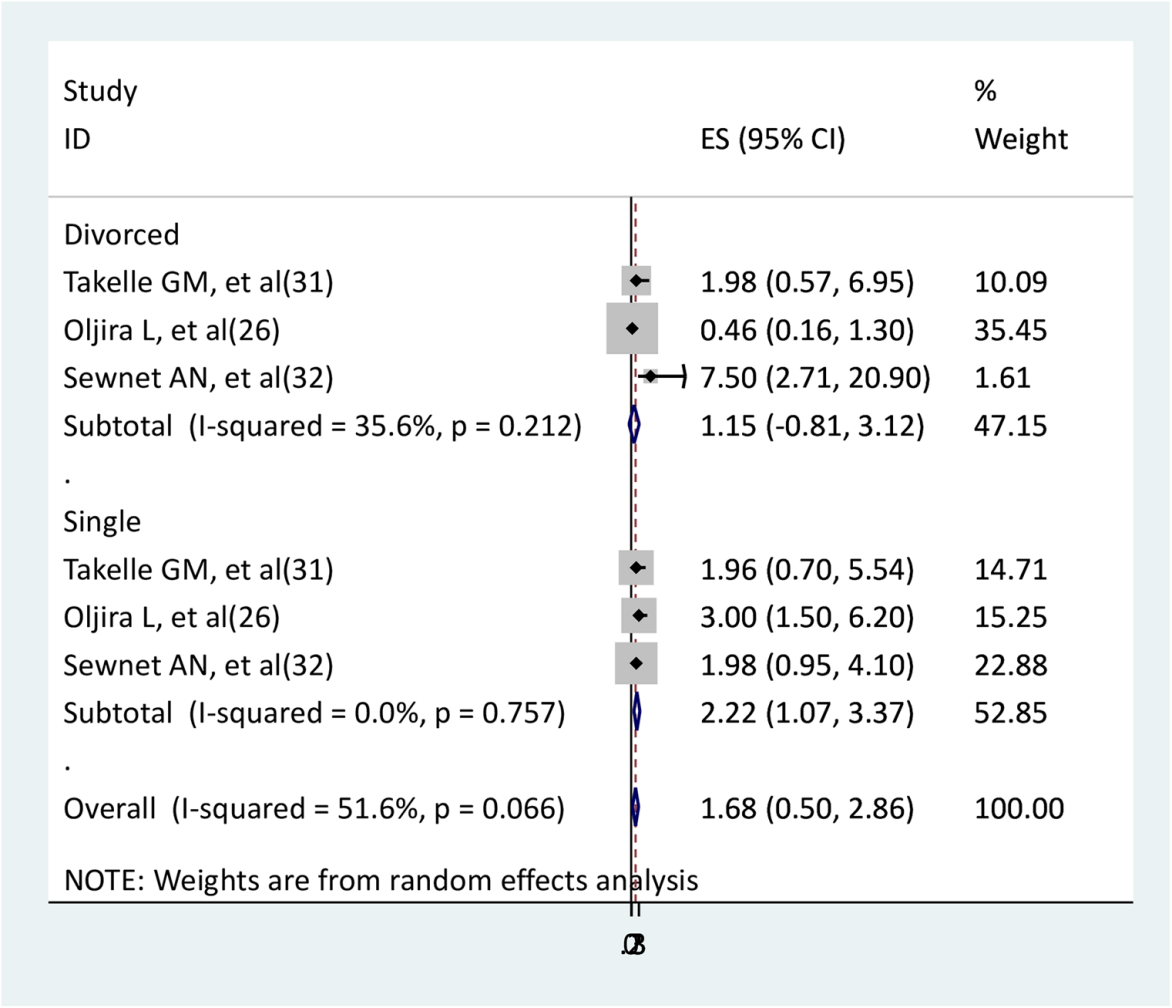
The association between marital status and depression.

The relationship between marital status and depression was determined using three studies (26, 31, 32). In the relationship between divorced pregnant women and depression, two studies (26, 31) did not show statistically significant association, but one study showed significant (32). In the relationship between single marital status and depression, one study showed a significant association (26), but two studies did not show (31, 32). The findings of the meta-analysis showed that single pregnant women had higher depression than married pregnant women (AOR = 2.22, 95% CI: 1.07–3.37). However, divorced pregnant women did not have a significant association with depression compared to married pregnant women (AOR = 1.15, 95CI:-0.81–3.12) (Figure 7).

### The association between social support and depression

**Figure 8:**
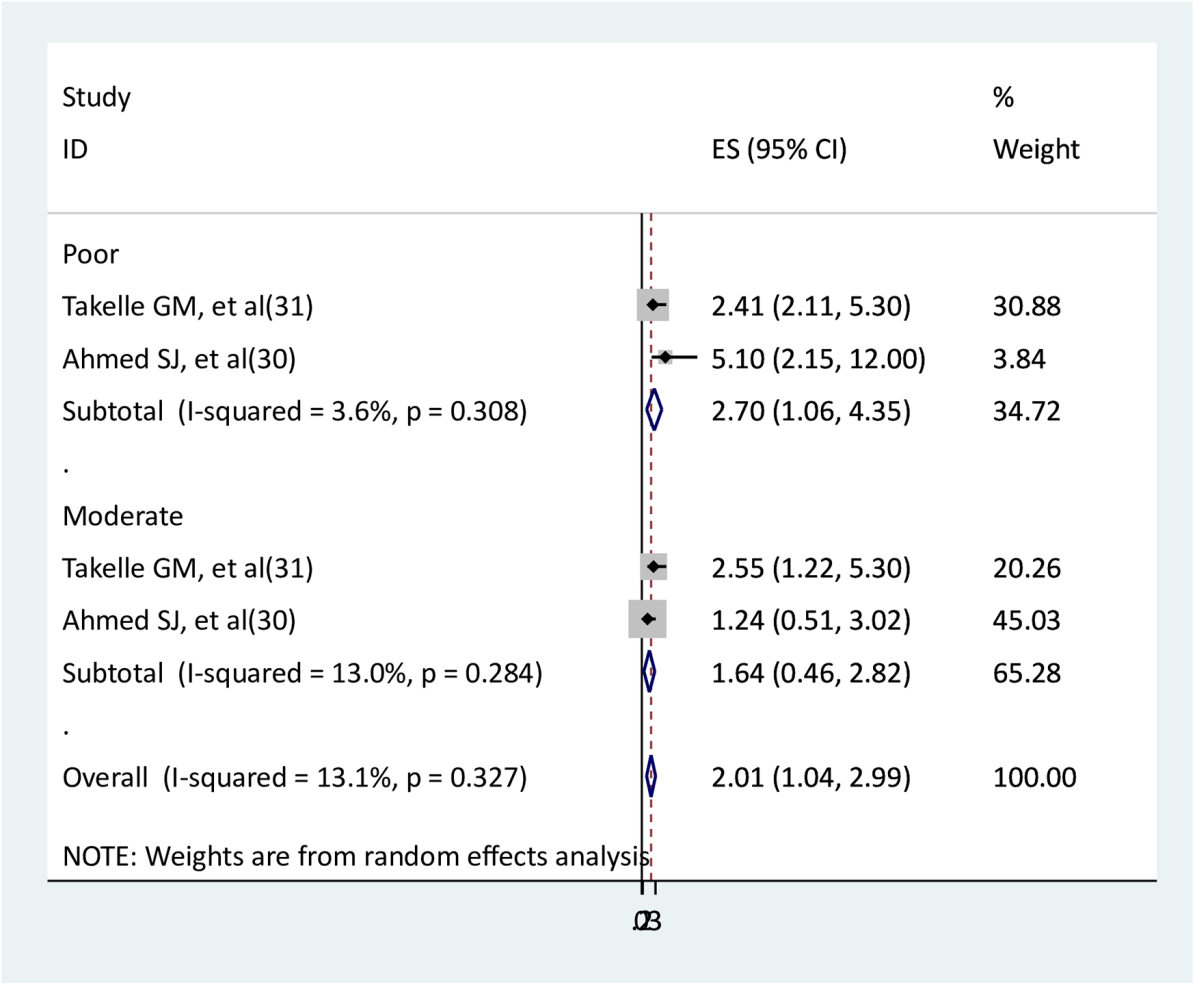
The association between social support and depression.

Two studies were used to evaluate the relationship between depression and social support (30, 31). Two studies that examined the relationship between depression and poor social support found statistically significant results. One study found statistical significance (31) in the relationship between depression and moderate social support, whereas the other found no statistical significance (30). The results of meta-analysis showed that pregnant women with poor social support had 2.7 times higher rates of depression than pregnant women with strong social support (AOR = 2.7, 95% CI: 1.06–4.35); however, pregnant women with moderate social support did not have a significant association with depression (AOR = 1.64, 95% CI: 0.46–2.82). Low heterogeneities between studies were observed in both poor and moderate social support (Figure 8).

### The association between intimate partner violence and depression

**Figure 9:**
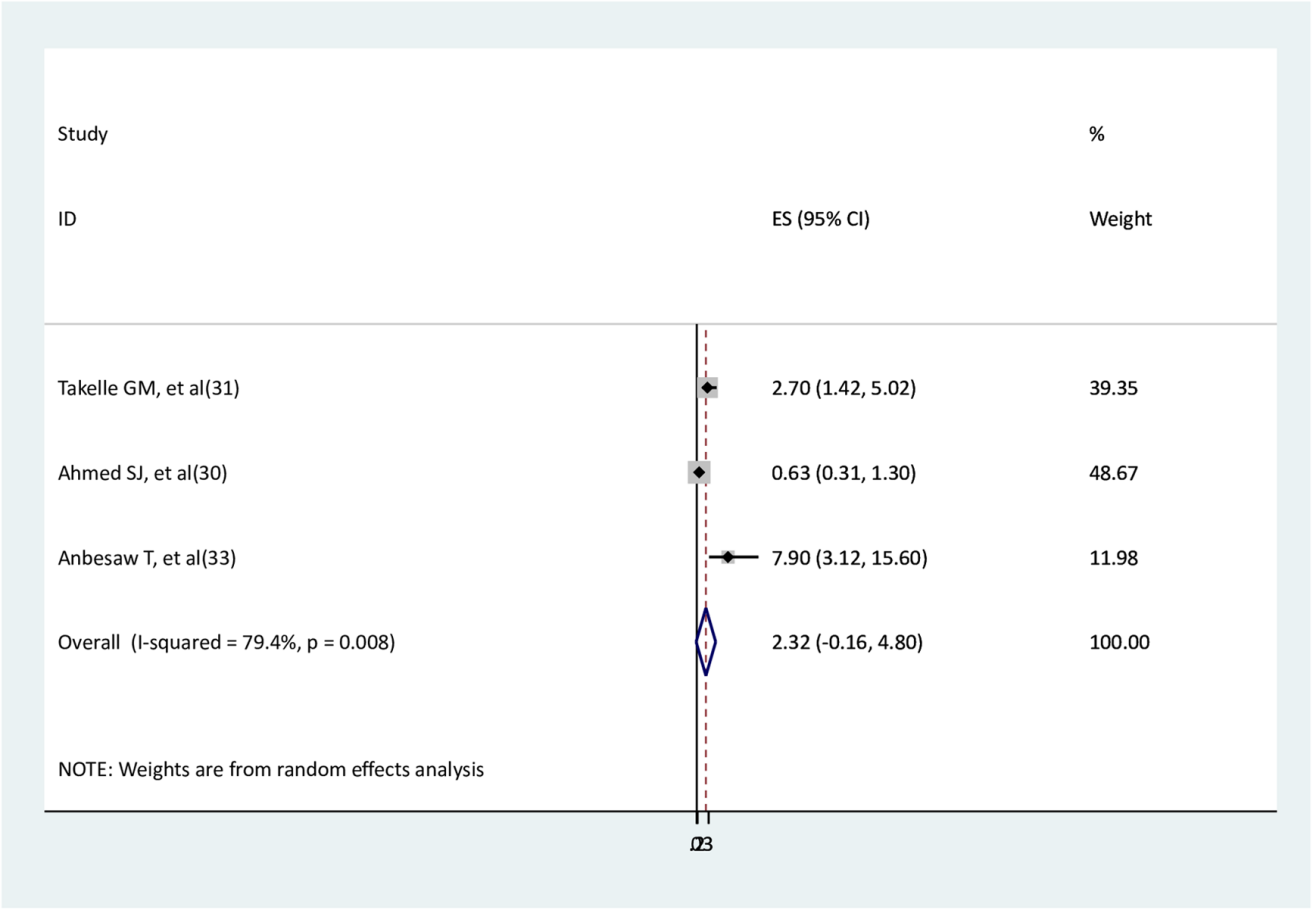
The association between intimate partner violate and depression.

Three studies (30, 31, 33) were used to assess the association between intimate partners and depression. Two studies (31, 33) found a statistical association, and the other one did not show a significant association (30). The findings of the meta-analysis revealed no significant association between intimate partner violence and depression (AOR = 2.32, 95% CI: −0.16–4.8), with substantial heterogeneity between the studies (Figure 9).

### The association between unplanned pregnancy and depression

**Figure 10:**
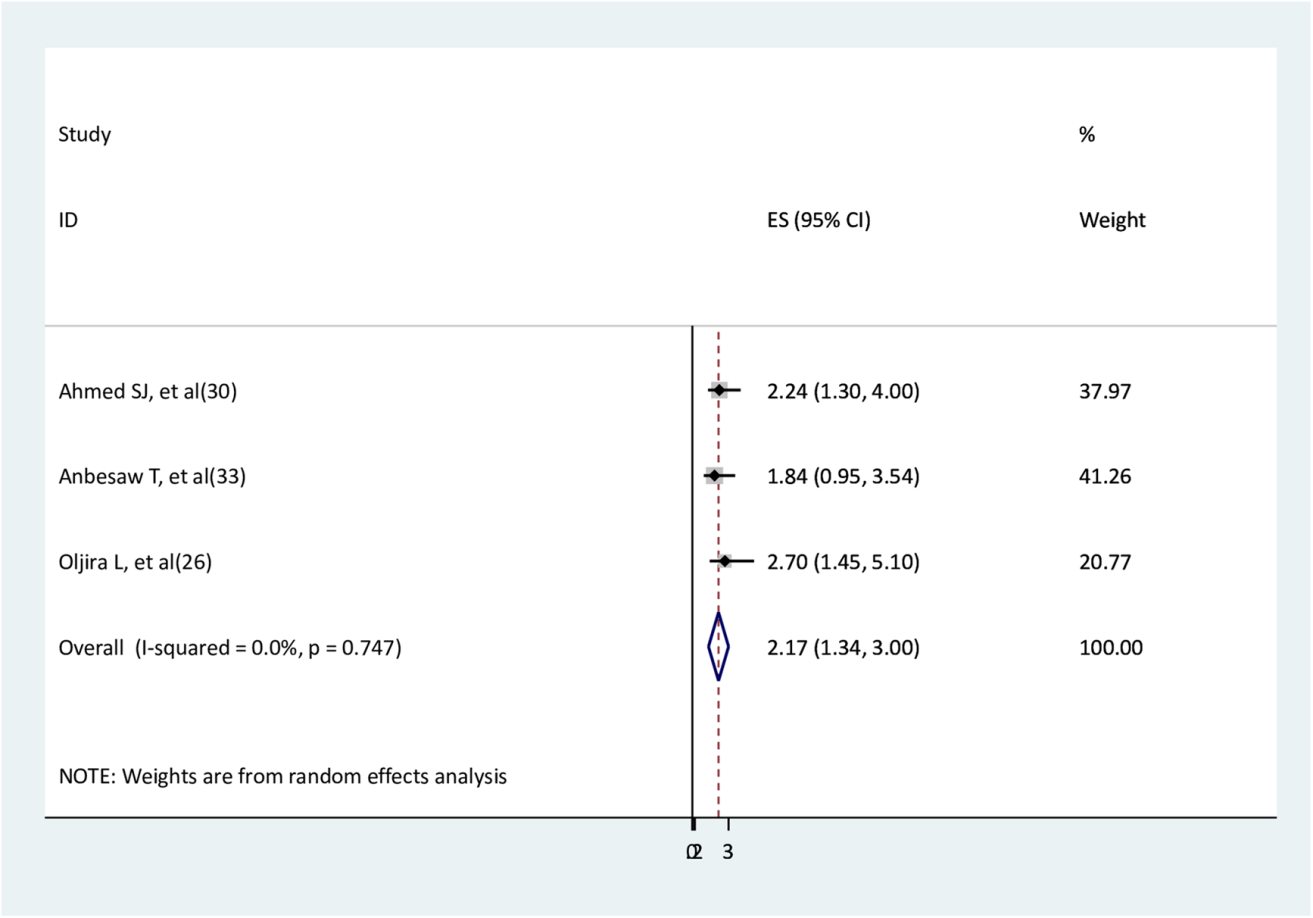
The association between unplanned pregnancy and depression.

The association between unplanned pregnancy and depression was determined using three studies (26, 30, 33). Two studies (26, 30) reported significant associations, and one study (31) reported a non-significant association. Women without planned pregnancies had 2.2 times higher depression than women with planned pregnancies (AOR = 2.17, 95% CI: 1.34–3.0) (Figure 10).

### The association between unsatisfied marital status and depression

**Figure 11:**
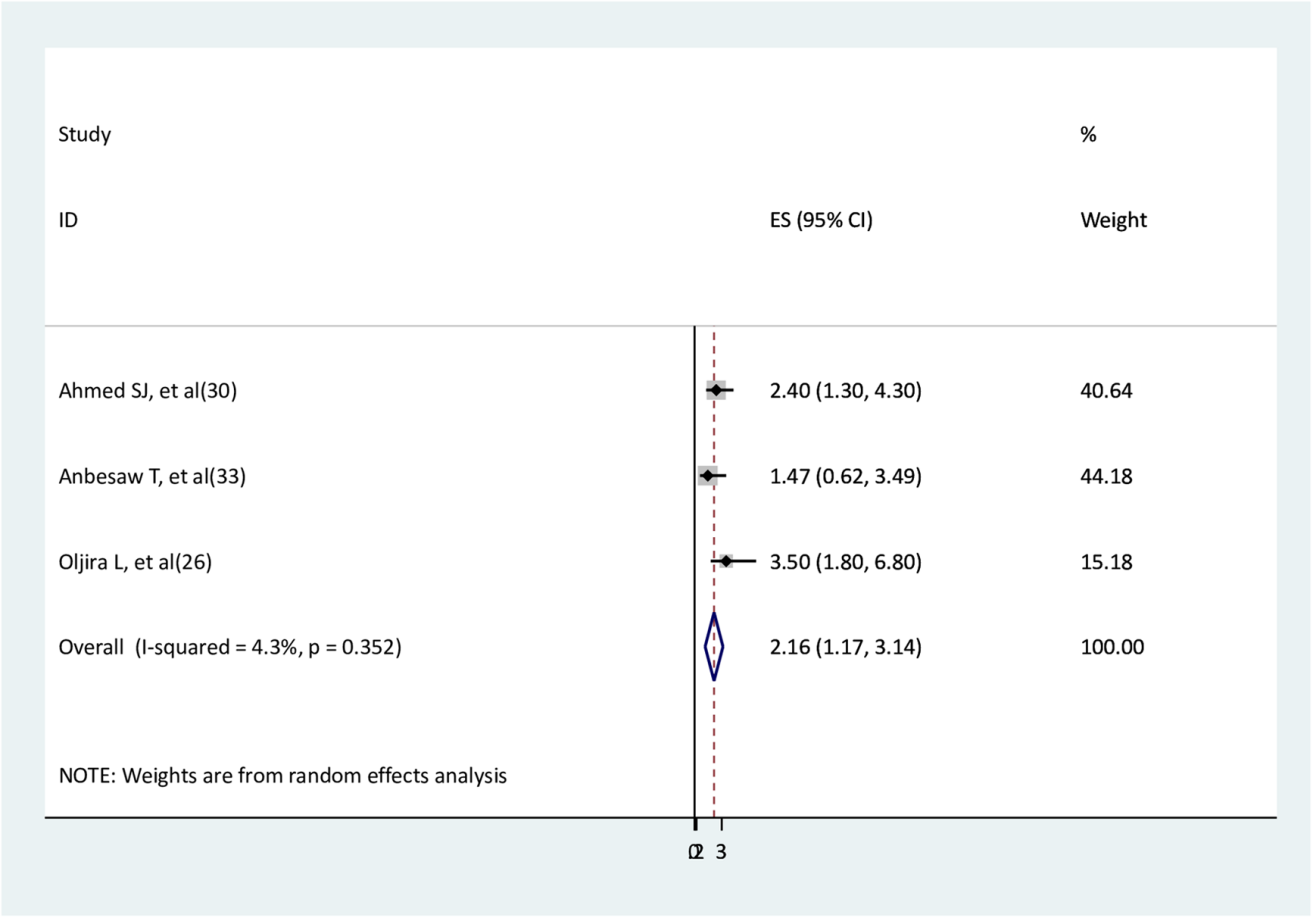
The association between unsatisfied marital status and depression.

Three studies (26, 30, 33) were used to assess the relationship between unsatisfied marital status and depression. The findings of two studies (26, 30) showed significant association, while the findings of one study (33) did not show. The findings of the meta-analysis revealed that pregnant women with unsatisfied marital status had two times higher depression than pregnant women with satisfied marital status (AOR = 2.16, 95% CI: 1.17–3.14) (Figure 11).

## Risk factors for anxiety

### The association between intimate partner violence and anxiety

**Figure 12:**
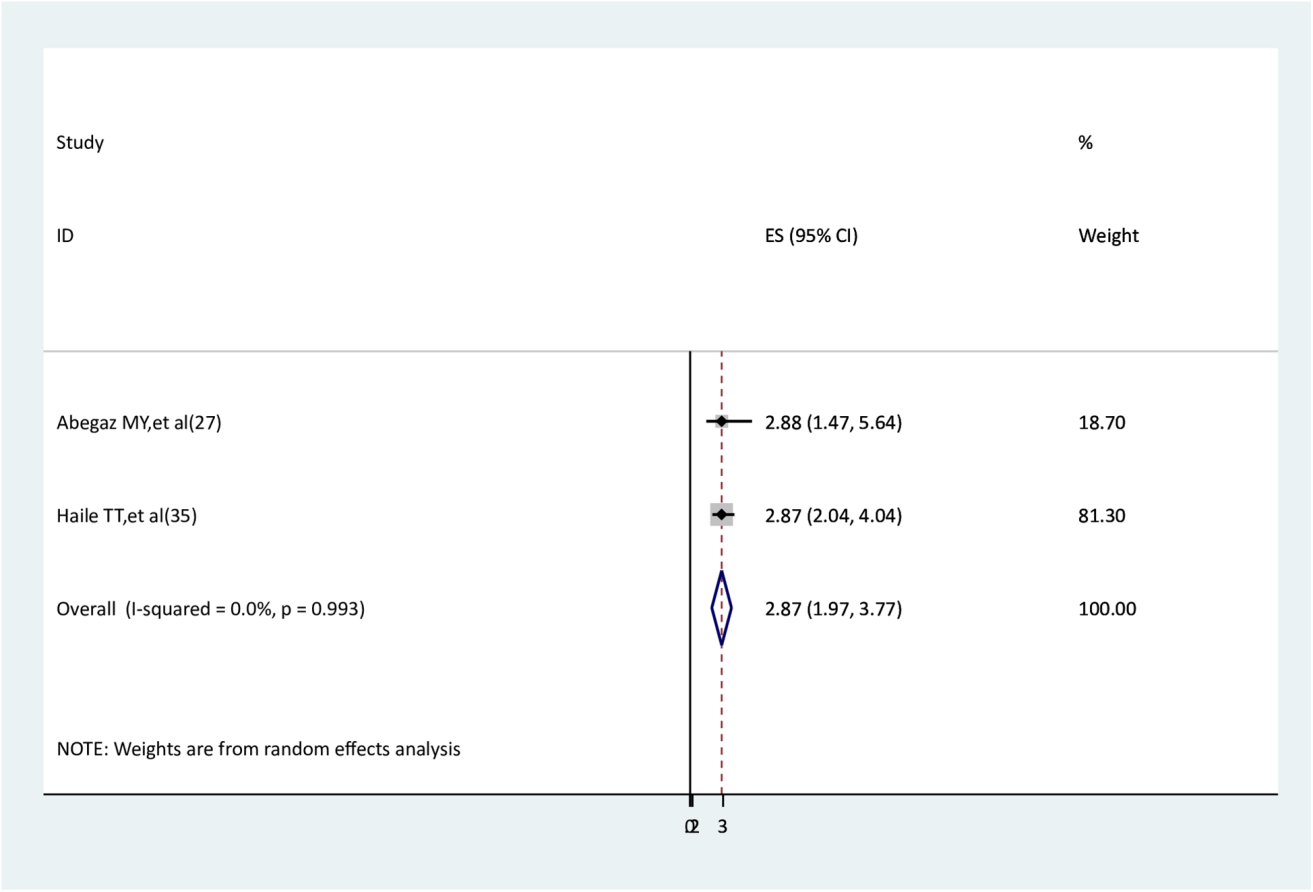
The association between intimate partner violence and depression.

The association between intimate partner violence and anxiety was examined by two studies (27, 29). Both studies found a statistically significant association. The findings of the meta-analysis showed that pregnant women who experienced intimate partner violence had higher anxiety than pregnant women who did not (AOR = 2.87, 95% CI: 1.97–3.77) (Figure 12).

### The association between social support and anxiety

**Figure 13:**
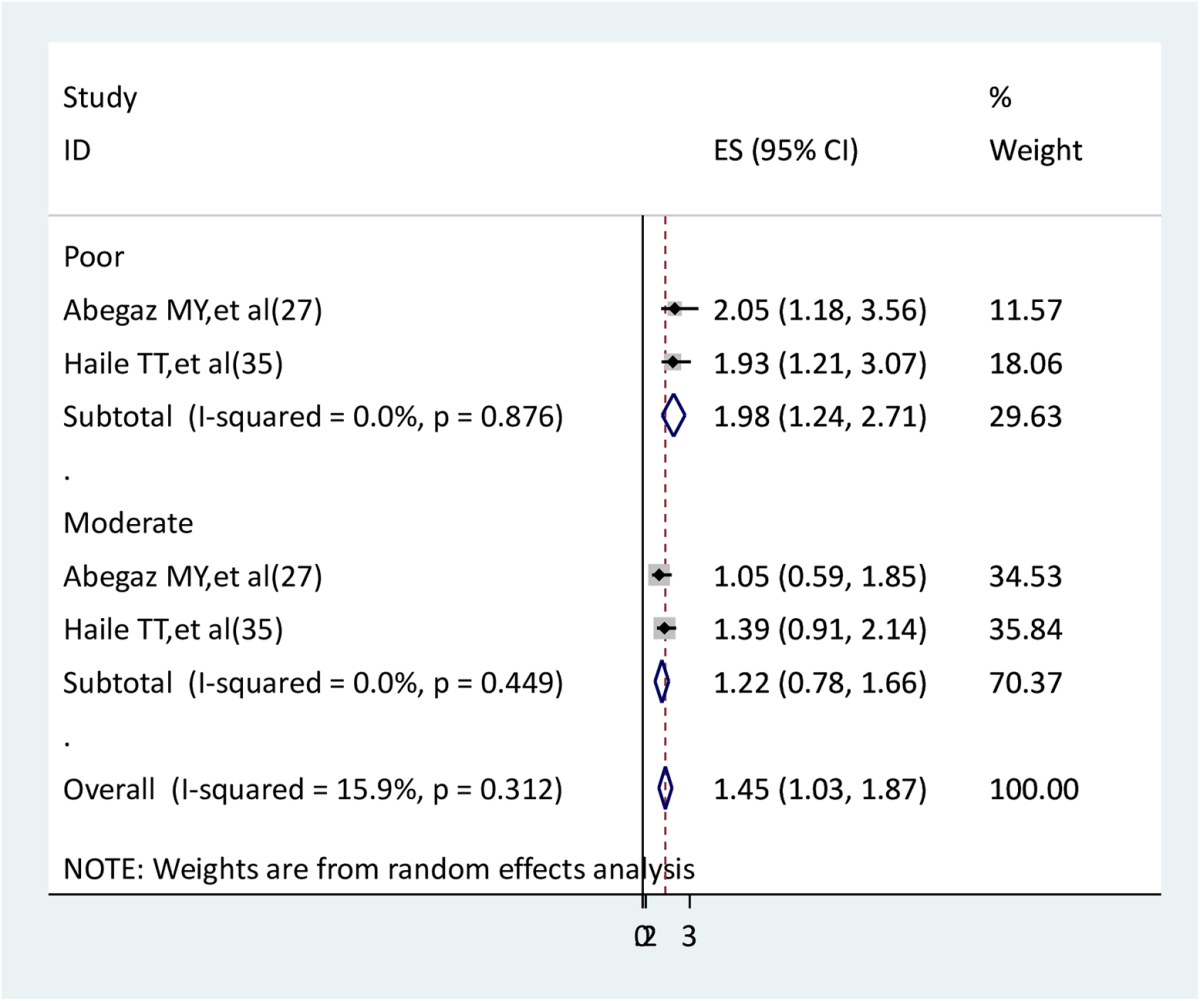
The association between social support and anxiety.

Two studies were identified to determine the relationship between social support and anxiety (27, 29). In the findings of two studies, there is a statistically significant association between poor social support and anxiety but not between moderate social support and anxiety. The results of the meta-analysis indicated that pregnant women with poor social support had nearly two times higher anxiety than pregnant women with strong social support (AOR = 1.98, 95% CI: 1.24–2.71). Pregnant women with moderate social support did not have a statistically significant association with anxiety (AOR = 1.22, 95% CI: 0.78–1.66). There was no heterogeneity between studies (Figure 13).

### History of medical illness and anxiety

**Figure 14:**
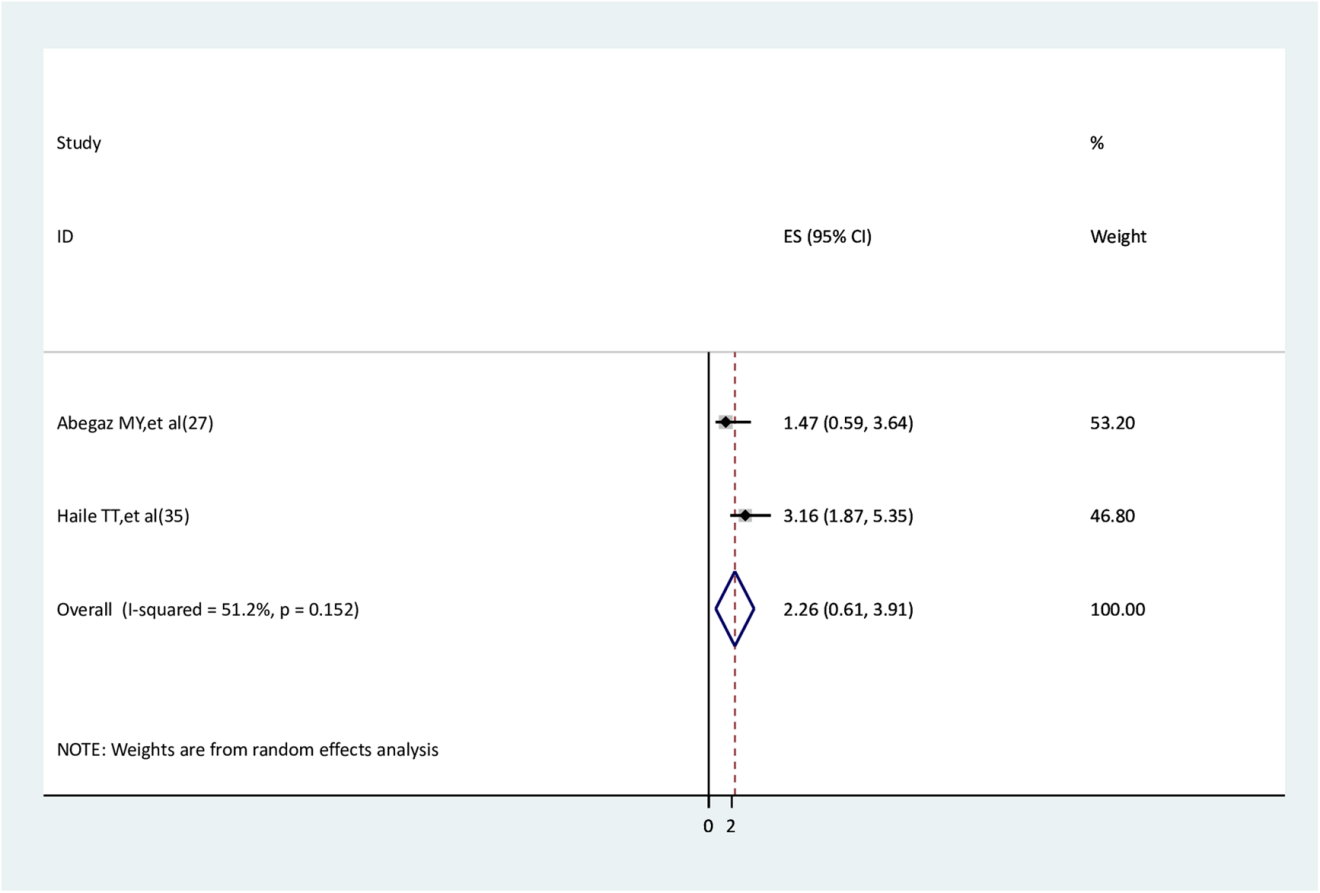
The association between history of medical illness and anxiety.

The association between a history of medical illness and anxiety was assessed using two studies (27, 29). One study had a significant association (29), but the other one did not (27). The results of the meta-analysis did not find a significant association between a history of medical illness and anxiety (AOR = 2.26, 95% CI: 0.61–3.91) (Figure 14).

## Discussion

Understanding COVID-19 pandemic effects on the psychological well-being of pregnant women is essential to preventing negative impacts and unexpected consequences for the mother and the fetus (4). Thus, the aim of this systematic review was to determine the pooled prevalence of depression and anxiety during the COVID-19 pandemic in Ethiopia.

In this systematic review, the pooled prevalence of depression among pregnant women during COVID-19 pandemic in Ethiopia was 24.7%. This magnitude is consistent with previous studies conducted at the global level (5), in Africa (6), and in Ethiopia (8–11). We did not find any differences in the pooled prevalence of depression in the present and the prior studies that did not take into account the pandemic in Ethiopia (9, 10). The World Health Organization discovered that the COVID-19 pandemic impacted mental health outcomes for women during and after pregnancy (35); therefore, this discovery was unexpected. The current depression magnitude, however, is lower than that of a previous study conducted globally (4) but higher than that of a previous study conducted in Kenya (7). This could be explained by the role of chance because of the small sample size, which makes it difficult to accurately detect the outcome of a single institute study conducted in Kenya. Other factors that may contribute to the variation in depression magnitude include societal level, social networks, genetics, cultural disparities (16, 17, 19), and variations in income levels (12–14). In the present meta-analysis, the pooled prevalence of anxiety among pregnant women after the onset of COVID-19 was 35.19%, which is higher than the previous studies conducted at the global level (5) and Kenya (7). This discrepancy may result from variations in cultural norms and income levels (12–14, 16, 17, 19). Moreover, there was a slightly higher magnitude of anxiety in the current study compared to a previous study in Ethiopia (11). This could be due to differences in how the social distancing strategy was implemented across Ethiopia during the COVID-19 epidemic, which could have an impact on the rise in anxiety symptoms (15).

The prevalence of depression was 26% and 24% in the Oromia and Amhara regions, respectively. This slight difference in the magnitude of depression between Oromia and Amhara region could be variations of genetic, societal (19) and financial levels (12–14) among study participants. The other explanation could be a sample size difference. A higher prevalence of depression was observed in 2021 at 26%, and then slightly decreased to 23% in 2022 by 3%. This may be because the COVID-19 immunization reduced COVID-19 mortality from 2020 to 2022 (36). Using DASS measurement tools, the prevalence of depression was 33%, while using EPDS and PHQ-9, the prevalence of depression was 23% and 14%, respectively. This variation could be a difference in the effectiveness of these tools in the diagnosis of depression.

Single and unsatisfied marital statuses were risk factors for depression. These findings were in line with the previous findings that reported that non-married and dissatisfied marital status had higher depression symptoms (30, 37). Unplanned pregnancy was a cause for depression. This evidence is consistent with a previous study done by Muskens L, et al. (38) that stated that women with an unplanned pregnancy were more vulnerable to developing depression. Pregnant women with poor social support had a higher depression and anxiety than pregnant women with strong social support. This finding was in line with a previous study (18). It might be attributed to having a poor social network (19), which is possibly based on increased social distance (15), which in turn worsens feelings of depression and anxiety. Pregnant women with intimate partner violence were more likely to have anxiety than pregnant women without intimate partners. This finding was in line with a previous study conducted by Wu F. et al. (39), which stated that intimate partner violence was significantly associated with anxiety.

All of the included studies were cross-sectional and, hence, had a lower level of evidence compared to other analytical studies. This systematic review only covered a small number of regions, making it impossible to generalize to the remaining regions. For the pooled prevalence of depression, heterogeneities were observed only in the Amhara region and in the study year 2021. Therefore, the heterogeneity in this region and study period should require further investigation. For other subgroup analyses, there was no heterogeneity. In general, the number of included studies was insufficient, resulting in inadequate power to detect the outcome in each subgroup. For the pooled prevalence of anxiety, subgroup analyses were not generally conducted as a result of the existence of few studies per category (one or two per category). Small numbers of studies were used to assess the association of risk factors with depression and anxiety, which led to insufficient evidence. Therefore, these results should be interpreted with caution, considering the small number of studies included, leading to the inadequate power of the study and its inability to detect the outcome accurately. However, this study found that the high rate of depression and anxiety in pregnant women during the COVID-19 pandemic in Ethiopia had significant implications for the mental and physical health of the woman as well as the wellbeing of her fetus. It is vital, therefore, to address them as a public health priority.

## Conclusion

One-fourth and nearly one-third pregnant women developed depression and anxiety, respectively, during the COVID-19 pandemic in Ethiopia. As a result, action is needed to incorporate perinatal mental health into policy and integrate mental health services, such as those for anxiety and depression, into routine prenatal care. Pregnant women with single and unsatisfied marital status required early antenatal screening and treatment to reduce depression. Unplanned pregnancy should also be given special attention. Poor social support is a cause of depression and anxiety. Pregnant women with violent intimate partners were more likely to develop anxiety.

## Data Availability

The original contributions presented in the study are included in the article/Supplementary Material, further inquiries can be directed to the corresponding author.
