# Supplementary material for: Depression and anxiety among pregnant women during COVID 19 pandemic in Ethiopia: a systematic review and meta-analysis
Source: Front Glob Womens Health. 2024 Dec 3;5:1453157. doi: 10.3389/fgwh.2024.1453157 (PMC11649664; doi:10.3389/fgwh.2024.1453157)
Supplement: Supplementary file 2 [file Table2.docx]

| S2 file searching strategies for depression and anxiety | | |
| --- | --- | --- |
| **Databases** |  | **Search terms** |
| Web of Science | **For depression** | Any filed contains “Depression” AND subject contains exact phrase “Pregnant women” AND subject contains “Ethiopia” |
|  | total articles | 71 |
|  | **For anxiety** | Any filed contains “Anxiety” AND subject contains exact phrase “Pregnant women” AND subject contains “Ethiopia” |
|  | Total article | 62 |
|  | Filtered by | Article, English language, publication date (01/01/2020-06/17/2024) |
| Science direct | **For depression** | Depression "Pregnant women" Ethiopia |
|  | Total article | 84 |
|  | **For anxiety** | Anxiety "Pregnant women" Ethiopia |
|  | Total article | 73 |
|  | Refined by | Publication year (2020-2024),article type(research article), and access type(open access) |
| PubMed | **For depression** | ((((((((Depression[MeSH Terms]) OR (Depression)) OR ("Depressive Symptoms")) OR ("Depressive Symptom")) OR ("Symptom Depressive")) OR ("Emotional Depression")) OR ("Depression Emotional")) AND ((((("Pregnant Women"[MeSH Terms]) OR ("Pregnant Women")) OR ("Women Pregnant")) OR ("Pregnant Woman")) OR ("Woman Pregnant"))) AND ((Ethiopia[MeSH Terms]) OR (Ethiopia)) |
|  | Total article | 65 |
|  | **For anxiety** | (((((((((((Anxiety[MeSH Terms]) OR (Anxiety)) OR (Angst)) OR (Nervousness)) OR (Hypervigilance)) OR ("Social Anxiety")) OR ("Anxieties Social")) OR ("Anxiety Social")) OR ("Social Anxieties")) OR (Anxiousness)) AND ((((("Pregnant Women"[MeSH Terms]) OR ("Pregnant Women")) OR ("Women Pregnant")) OR ("Pregnant Woman")) OR ("Woman Pregnant"))) AND ((Ethiopia[MeSH Terms]) OR (Ethiopia)) |
|  | Total article | 27 |
|  | Filtered by | Free full text and publication year (01/01/2020-17/06/204) |
| Google scholar | **For depression** | Depression "pregnant women" Ethiopia |
|  | Total article | 99 |
|  | **For anxiety** | Anxiety "pregnant women" Ethiopia |
|  | Total articles | 80 |
|  | Filtered by | Publication year (2020-2024) and English language |
| African journals online | **For depression** | Depression "pregnant women" Ethiopia |
|  | Total article | 24 |
|  | **For anxiety** | Anxiety "pregnant women" Ethiopia |
|  | Total articles | 18 |
| **Total article for depression** | | 343 |
| **Total article for anxiety** | | 260 |
