# Supplementary material for: Depression and anxiety among pregnant women during COVID 19 pandemic in Ethiopia: a systematic review and meta-analysis
Source: Front Glob Womens Health. 2024 Dec 3;5:1453157. doi: 10.3389/fgwh.2024.1453157 (PMC11649664; doi:10.3389/fgwh.2024.1453157)
Supplement: Supplementary file 3 [file Table3.docx]

| Authors | study year | study region | study design | sample size | Cases (%) | Outcome | Measurement tools |
| --- | --- | --- | --- | --- | --- | --- | --- |
| Takelle GM, et al.(31) | 2022 | Amhara | Cross sectional | 473 | 91(19.2) | Depression | EPDS |
| Sewnet AN, et al (32) | 2021 | Amhara | Cross sectional | 422 | 144(34.1) | Depression | DASS |
| Anbesaw T, et al (33) | 2021 | Amhara | Cross sectional | 451 | 84 (18.6) | Depression | EPDS |
| Seid J, et al (34) | 2022 | Amhara | Cross sectional | 552 | 178 (32.2) | Depression | DASS |
| Oljira L, et al (26) | 2022 | Oromia | Cross sectional | 370 | 62 (16.8) | Depression | EPDS |
| Ahmed SJ, et al(30) | 2021 | Oromia | Cross sectional | 329 | 109(33.1) | Depression | EPDS |
| Abegaz MY,et al(27) | 2021 | Amhara | Cross sectional | 408 | 115(28.2) | Depression | PHQ-9 |
|  |  |  |  |  | 179(43.9) | Anxiety | PRAQR |
| Tarafa H, et al (36) | 2021 | Oromia | Cross sectional | 406 | 121(29.8) | Depression | EPDS |
|  |  |  |  |  | 133(32.7) | Anxiety | PRAQR |
| Haile TT,et al (35) | 2021 | Amhara | Cross sectional | 858 | 96(11.2) | Depression | PHQ-9 |
|  |  |  |  |  | 252(29.4) | Anxiety | PRAQR |

S3 File. Extracted data for depression and anxiety
