# Supplementary material for: Depression and anxiety among pregnant women during COVID 19 pandemic in Ethiopia: a systematic review and meta-analysis
Source: Front Glob Womens Health. 2024 Dec 3;5:1453157. doi: 10.3389/fgwh.2024.1453157 (PMC11649664; doi:10.3389/fgwh.2024.1453157)
Supplement: Supplementary file 4 [file Table4.docx]

|  | S4 file quality assessment of included studies | | | | | | | | | |
| --- | --- | --- | --- | --- | --- | --- | --- | --- | --- | --- |
| Authors, Publication year | | Study outcome | Selection | | | | Comparability | Outcome | | Total score (%) |
|  |  |  | Representativeness* | Sample size* | Non-Response rate* | Ascertainment of the screening tool** | Confounding control** | Assessment of the outcome** | Statistical test:* |  |
| Takelle GM, et al.(31) | | Depression | * | * | * | ** | ** | ** | * | 10/10 (100%) |
| Sewnet AN, et al (32) | | Depression | * | * | ⁎ | ⁎⁎ | ** | ** | ⁎ | 10/10(100%) |
| Ahmed SJ, et al(30) | | Depression | ⁎ | ⁎ | ⁎ | ⁎⁎ | ** | ** | * | 10/10 (100%) |
| Anbesaw T, et al (33) | | Depression | * | * | * | ** | ** | ** | * | 10/10 (100%) |
| Seid J, et al (34) | | Depression | * | * | * | ** | ** | ** | * | 10/10 (100%) |
| Oljira L, et al (26) | | Depression | * | * | * | ** | ** | ** | * | 10/10 (100%) |
| Abegaz MY,et al(27) | | Depression | * | * | * | ** |  | ** |  | 7/10(70%) |
| Tarafa H, et al (36) | | Depression | * | * | * | ** |  | ** |  | 7/10(70%) |
| Haile TT,et al (35) | | Depression | * | * | * | ** |  | ** |  | 7/10(70%) |
| Abegaz MY,et al(27) | | Anxiety | * | * | * | ** | ** | ** | * | 10/10(100%) |
| Tarafa H, et al (36) | | Anxiety | * | * | * | ** | ** | ** | ** | 10/10=100% |
| Haile TT,et al (35) | | Anxiety | * | * | * | ** | ** | ** | * | 10/10(100%) |

The annotation of one asterisk (*) had 1 value/score, while the annotation of two asterisks (**) had 2 value/score.

**NEWCASTLE - OTTAWA QUALITY ASSESSMENT SCALE (adapted for cross sectional studies)**

**Selection:** (Maximum 5 scores)

**1) Representativeness of the cases:**

a) Truly representative of the HCC patients (consecutive or random sampling of cases). 1 score

b) Somewhat representative of the average in the HCC patients (non-random sampling) . 1 score

c) Selected demographic group of users. 0 score

d) No description of the sampling strategy. 0 score

**2)** **Sample size:**

a) Justified and satisfactory (≥ 400 HCC included). 1 score

b) Not justified (<400 HCC patients included). 0 score

3) **Non-Response rate**

a) The response rate is satisfactory (≥95%). 1 Score

b) The response rate is unsatisfactory (<95%), or no description. 0 Score

4) **Ascertainment of the screening/surveillance tool**:

a) Validated screening/surveillance tool. 2 scores

b) Non-validated screening/surveillance tool, but the tool is available or described. 1 score

c) No description of the measurement tool. 0 score

**Comparability: (Maximum 2 stars)**

1) The potential confounders were investigated by subgroup analysis or multivariable analysis.

a) The study investigates potential confounders. 2 score

b) The study does not investigate potential confounders. 0 score

**Outcome: (Maximum 3 stars)**

1) **Assessment of the outcome:**

a) Independent blind assessment. 2 scores

b) Record linkage. 2 scores

c) Self report. 1 score

d) No description. 0 score

2) **Statistical test:**

a) The statistical test used to analyze the data is clearly described and appropriate. 1 score

b) The statistical test is not appropriate, not described or incomplete. 0 score
